# Diamine Oxidase and Gastrointestinal Diseases

**DOI:** 10.3390/biom16081136

**Published:** 2026-08-05

**Authors:** Paulina Żybul, Adam Przybyłkowski, Oksana Wojas, Bolesław Samoliński

**Affiliations:** 1 Department of Gastroenterology and Internal Medicine, Medical University of Warsaw, 02-097 Warsaw, Poland; paulina.zybul@uckwum.pl (P.Ż.); adam.przybylkowski@wum.edu.pl (A.P.); 2 Department of Prevention of Environmental Hazards Allergology and Immunology, Medical University of Warsaw, 02-097 Warsaw, Poland; boleslaw.samolinski@wum.edu.pl

**Keywords:** diamine oxidase, histamine metabolism, intestinal barrier, gastrointestinal diseases

## Abstract

Histamine plays an essential role in gastrointestinal physiology and immune control, while its balance in the intestinal environment is largely maintained through degradation by diamine oxidase (DAO), a copper-dependent enzyme mainly produced by differentiated enterocytes. Impaired DAO activity reduces the intestinal barrier to luminal histamine, leading to increased local and systemic exposure and contributing to altered motility, permeability, visceral sensitivity, and immune activation. Accumulating research suggests that disturbances in diamine oxidase regulation contribute to the development of various gastrointestinal diseases, including histamine intolerance, inflammatory and functional bowel disorders, intestinal ischemia, and liver pathology. This review summarizes current knowledge on the biochemistry, localization, and regulation of DAO, as well as histamine signalling via H1–H4 receptors in the gastrointestinal tract. We highlight the interaction between epithelial injury, immune activation, dysbiosis, and histamine accumulation, and discuss the divergent interpretation of mucosal versus circulating DAO activity. Finally, we critically assess the diagnostic limitations of serum DAO and explore emerging therapeutic strategies targeting impaired histamine degradation. Although DAO represents a promising adjunctive biomarker and therapeutic target, its clinical utility requires standardized measurement methods and integrative diagnostic approaches.

## 1. Introduction

Histamine is a pleiotropic biogenic amine involved in a wide range of physiological processes, including immune regulation, control of gastric acid secretion, modulation of vascular tone, neurotransmission, and IgE-mediated allergic responses. It is synthesized from the amino acid histidine via enzymatic decarboxylation in multiple cell types, including mast cells, basophils, and enterochromaffin cells of the gastrointestinal tract [1,2]. Given that excessive histamine signalling may disturb both mucosal integrity and systemic homeostasis, its clearance is stringently controlled by two principal metabolic pathways: intracellular degradation mediated by histamine N-methyltransferase (HNMT), and extracellular oxidative deamination catalyzed by diamine oxidase (DAO) [3,4,5,6]. DAO exhibits high activity in the intestinal mucosa and is predominantly localized within the cytoplasm of mature enterocytes [7,8]. Reduced mucosal DAO activity has been associated with epithelial injury and impaired intestinal barrier integrity in both experimental models and human intestinal disease [9,10]. Emerging evidence associates diminished DAO activity with a range of gastrointestinal disorders. Impaired enzymatic degradation of histamine within the intestinal lumen may result in elevated local and systemic histamine concentrations, evoking changes in motility, mucosal permeability, visceral sensitivity, and immune activation [8,11,12,13,14,15]. Accordingly, circulating DAO has been proposed as a potential biomarker of intestinal mucosal integrity as well as a therapeutic target for histamine-related gastrointestinal dysfunction [16,17,18,19,20]. Despite increasing recognition of the clinical relevance of DAO, its role in gastrointestinal disorders remains incompletely characterized. Although DAO is most often discussed in the context of histamine intolerance (HI), the clinical implications of altered DAO activity for gastrointestinal pathology are still not fully understood. Fundamental knowledge gaps persist regarding its distribution along the gastrointestinal tract and the extent to which impaired enzymatic degradation of histamine contributes to mucosal dysfunction. This review seeks to synthesize current evidence on the physiological role of DAO in the gut and its relevance to clinical gastroenterology, with particular emphasis on its potential diagnostic utility and emerging therapeutic strategies targeting defective histamine degradation.

## 2. Biochemistry of Diamine Oxidase (DAO)

**Figure 1:**
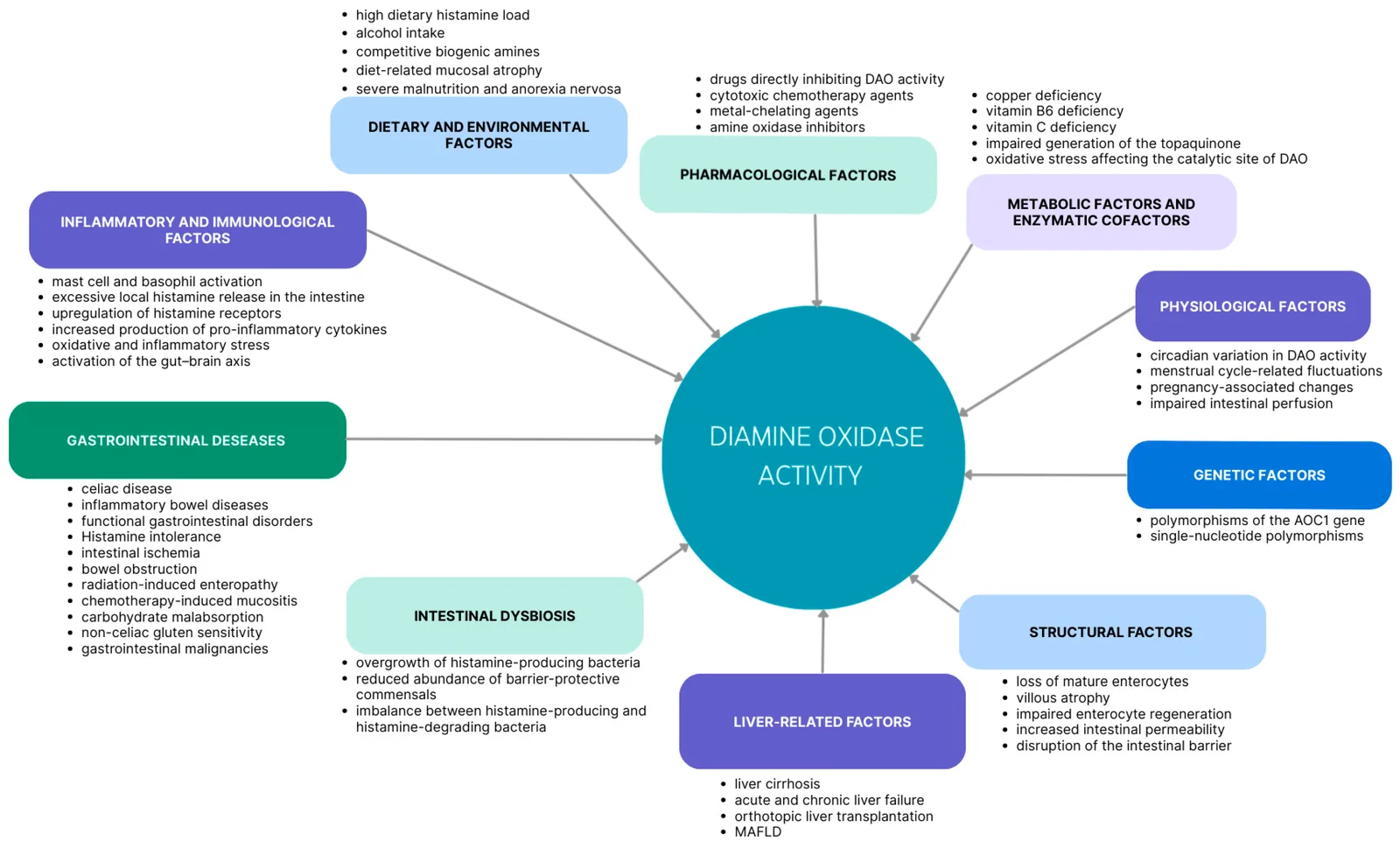
Overview of factors influencing diamine oxidase (DAO) activity.

Diamine oxidase (DAO; EC 1.4.3.22), encoded by the AOC1 gene, is a copper-dependent amine oxidase that catalyzes the conversion of histamine into imidazole-4-acetaldehyde, hydrogen peroxide and ammonia. Beyond histamine, DAO also metabolizes other structurally related biogenic diamines, including putrescine and cadaverine [1,21,22]. As a predominantly extracellular, secreted enzyme, DAO constitutes a principal component of the intestinal mucosal barrier by limiting the transepithelial transfer of luminal histamine into the systemic circulation [12,23,24]. DAO activity relies on copper ions coordinated within the catalytic site and the topaquinone (TPQ) cofactor, which is formed through post-translational oxidation of a conserved tyrosine residue [25,26]. Its functional capacity is influenced by multiple factors, including epithelial integrity, oxidative and inflammatory stress, as well as exposure to pharmacological agents such as amine oxidase inhibitors, metal chelators, or cytostatic drugs [17,27,28,29]. Nutritional factors, particularly the luminal burden of biogenic amines and microbiota-derived metabolites, may also modulate DAO activity [4,24,30]. Since DAO is synthesized and secreted by mature enterocytes, conditions that disrupt mucosal architecture or impair enterocyte viability are typically associated with diminished enzymatic activity [6,8,9,29]. In contrast to DAO, which governs extracellular histamine catabolism, histamine N-methyltransferase (HNMT) functions intracellularly, primarily in tissues with low DAO expression [31,32]. Together, these pathways ensure comprehensive histamine clearance, with DAO serving as the primary defence against excessive luminal or dietary histamine within the gastrointestinal tract.The major determinants of DAO activity are summarized in Figure 1.

## 3. Histamine Signalling in the Gastrointestinal Tract

Within the gastrointestinal tract, histamine exerts a broad spectrum of biological effects that are mediated predominantly by four distinct G-protein-coupled receptor subtypes (H1–H4), each characterized by specific patterns of expression and specialized functional roles [33,34]. Within the gut, H1 receptors are abundantly expressed on smooth muscle cells and sensory neurons, where their activation promotes muscle contraction, enhances vascular permeability, and stimulates sensory nerve signalling. Collectively, these effects contribute to typical gastrointestinal manifestations, including abdominal pain, cramping, diarrhea and visceral hypersensitivity [35,36,37]. H2 receptors, primarily expressed on parietal cells and the microvasculature, regulate gastric acid secretion and local vasodilatation, contributing to dyspeptic symptoms and potentially aggravating reflux-related disorders when overstimulated [38,39,40,41]. H3 receptors, predominantly presynaptic within the enteric nervous system, modulate neurotransmitter release and influence intestinal motility, while H4 receptors, mainly expressed on mucosal immune cells, contribute to chemotactic processes and potentiate local inflammatory responses, particularly those associated with Th2-driven immunity [42,43,44,45,46,47]. Together, these receptors orchestrate the gastrointestinal response to luminal histamine. Impaired DAO activity may lead to augmented receptor-mediated effects, manifesting as postprandial abdominal discomfort, bloating, diarrhea, and increased visceral sensitivity [24,48,49,50]. Beyond their physiological functions, histamine receptor isoforms also contribute to the pathogenesis of gastrointestinal diseases. H1 receptor-mediated signalling has been implicated in visceral hypersensitivity in irritable bowel syndrome, whereas H4 receptor activation has been implicated in immune cell recruitment and inflammatory responses in inflammatory bowel disease and gastrointestinal allergy [37,46,47].

## 4. Localization of Diamine Oxidase

DAO exhibits marked tissue specificity, although its distribution varies among mammalian species. In both humans and rats, high DAO activity has been demonstrated in the small intestinal mucosa, while DAO has also been identified in the placenta and kidneys in humans [7,51,52,53]. In humans, DAO is predominantly localized in the cytoplasm of mature enterocytes of the small and large intestine, with little or no expression in crypt epithelial cells [7,10]. Recent immunohistochemical analysis has further demonstrated that DAO is distributed throughout the upper gastrointestinal tract, with the strongest immunostaining observed in the subepithelial lamina propria, while lower staining intensity is present in the epithelium and deeper lamina propria. DAO immunoreactivity has also been detected in intracellular and extracellular compartments as well as in vascular endothelial cells [54]. Experimental studies in rats have shown that intestinal DAO activity increases towards the distal small intestine, reaching the highest levels in the distal jejunum and ileum, and that the enzyme is localized predominantly within the cytosol and organelle fractions of enterocytes, with only minimal association with the brush-border membrane [51]..Similarly, it has been demonstrated that, in the rat intestine, DAO is concentrated within mature villus enterocytes rather than crypt cells and is largely absent from the brush-border membrane, supporting its intracellular localization [8]. Although these findings provide important insights into intestinal DAO biology, species-specific differences in DAO expression and tissue distribution should be considered when extrapolating experimental data to humans [8]. Studies using polarized Caco-2 monolayers together with human small intestinal tissue demonstrated that DAO is secreted in a polarized manner predominantly through the basolateral membrane and localized mainly along the lateral and basal surfaces of villus enterocytes. Heparin markedly enhanced basolateral DAO release, indicating that intestinal DAO secretion is dynamically regulated [55]. These findings indicate that DAO is predominantly produced by differentiated villus enterocytes and is strategically positioned at the epithelial-stromal interface, where it facilitates extracellular degradation of histamine before its entry into the systemic circulation. More recently, studies have demonstrated that extracellular DAO can bind to cell-surface heparan sulphate proteoglycans, undergo cellular internalization and intracellular trafficking, suggesting additional biological functions beyond extracellular histamine degradation [56].

## 5. Histamine Intolerance (HIT)

Histamine intolerance (HIT) represents a clinically appealing but diagnostically challenging construct, in which heterogeneous gastrointestinal and extraintestinal symptoms are attributed to impaired histamine degradation rather than to a clearly defined disease entity. Because increased circulating histamine may activate all histamine receptor subtypes (H1–H4) distributed throughout the body, the resulting symptom profile is non-specific and frequently overlaps with allergic conditions, functional gastrointestinal disorders, and other food-related hypersensitivity reactions [6,49]. In this context, HIT is not classified as a primary gastrointestinal disease, although it manifests predominantly with gastrointestinal complaints, and reduced intestinal diamine oxidase (DAO) activity is commonly considered central to its pathophysiology [50]. From a mechanistic perspective, HIT is commonly described as a state in which intestinal histamine exposure (dietary or endogenously generated) exceeds local enzymatic degradation capacity, allowing histamine to accumulate at the mucosal interface. Several studies report that patients presenting with symptoms attributed to HIT exhibit lower plasma DAO activity [57,58]. Clinically, the symptom spectrum associated with HIT is broad; bloating, postprandial fullness, diarrhea, abdominal pain and constipation are among the most frequently reported complaints in patients diagnosed with histamine intolerance [50]. However, these manifestations remain largely non-specific and may mimic allergic reactions including pruritus, asthmatic symptoms or headaches, with gastrointestinal symptoms generally predominating [49]. Although the key role of histamine excess and reduced DAO activity is supported by clinical observations showing symptom improvement following a low-histamine diet and DAO supplementation [6,48,59,60,61], accurate diagnosis of HIT remains problematic. Diagnostic evaluation requires careful exclusion of other gastrointestinal and systemic conditions with overlapping presentations, allowing symptoms to be attributed to histamine overload rather than alternative etiologies [57,62,63]. Clinical suspicion is usually raised when symptoms reproducibly follow consumption of foods known to accumulate histamine during processing or storage, while individual tolerance thresholds vary substantially [64,65]. At the intestinal level, insufficient DAO activity may permit even physiologically normal amounts of dietary histamine to enter the circulation and induce symptoms, supporting the concept of DAO deficiency as a central mechanistic component of HIT [3,31].

Despite frequent clinical use, serum DAO provides limited diagnostic resolution. Its circulating activity should not be interpreted as a direct surrogate for mucosal DAO function at the epithelial barrier [63,66,67]. The interpretation of DAO measurements should also take into account methodological differences among studies, including variations in biological samples, assay methods, and the lack of standardized procedures for DAO determination. In addition, values are difficult to contextualize because reference intervals are not robustly established, physiological variability related to circadian rhythms, menstrual cycle or pregnancy is substantial, and studies have reported inconsistent relationships between serum DAO and symptom burden [31,49,57,68,69,70]. For these reasons, serum DAO may serve only as an adjunctive diagnostic tool and cannot be considered a standalone biomarker of histamine intolerance [6,49]. Diagnostic accuracy may be improved by evaluating clinical response to a low-histamine diet, excluding alternative causes of histamine excess (allergic, metabolic or toxic), and assessing intestinal DAO activity [49,57,63]. Analysis of DAO-related genetic polymorphisms may further complement the diagnostic work-up.

Because DAO deficiency is regarded as a principal mechanistic driver of histamine intolerance, factors that modulate its activity must be considered during evaluation. Numerous medications (including verapamil, clavulanic acid, isoniazid, cefuroxime, metoclopramide, diclofenac and amitriptyline) have been reported to reduce DAO activity, most likely through functional interference with histamine oxidation and/or competition for enzymatic capacity [1,71,72,73,74]. Similarly, other biogenic amines such as putrescine and cadaverine compete with histamine for DAO-mediated degradation, thereby reducing its barrier function. Deficiencies in enzymatic cofactors essential for DAO activity further impair catalytic capacity; accordingly, assessment of vitamin B6, vitamin C and copper status should be incorporated into diagnostic evaluation [1,31,71,75,76,77]. Alcohol represents an additional potent modulator, simultaneously promoting endogenous histamine release and impairing DAO-dependent catabolism [6,31]. Moreover, dysbiosis and increased abundance of histamine-producing bacteria may further burden the DAO system and exacerbate histamine accumulation [30]. Structural injury of the intestinal mucosa, as observed in conditions such as inflammatory bowel disease or celiac disease, is likewise associated with reduced mucosal DAO expression and increased intestinal permeability, facilitating systemic histamine translocation and symptom development [62,78,79]. Finally, genetic predisposition contributes to interindividual variability, as approximately 50 single-nucleotide polymorphisms within the AOC1 gene have been identified, several of which (including rs10156191, rs1049742, rs2268999) are associated with reduced DAO enzymatic activity [80,81,82,83,84]. Although several nutritional approaches have been proposed to support endogenous DAO function, current clinical evidence for their efficacy remains limited. Therefore, further well-designed clinical studies are required before such interventions can be recommended for the management of histamine intolerance [6,31,49].

## 6. DAO in Gastrointestinal Diseases

### 6.1. DAO and Histamine Metabolism in Inflammatory Bowel Diseases

Inflammatory bowel diseases (IBD), encompassing Crohn’s disease (CD) and ulcerative colitis (UC), comprise chronic immune-mediated disorders characterized by recurrent episodes of intestinal inflammation. Over recent decades, the global burden of IBD has risen substantially, a trend closely linked to progressive industrialization and the widespread adoption of Western lifestyle [85]. Multiple studies show that mucosal DAO levels are consistently reduced in active IBD, while findings regarding serum DAO remain inconsistent across reports. Local enzyme reduction correlates strongly with villous atrophy, epithelial destruction, inflammatory activity, and increased intestinal permeability [8,86,87]. Intestinal DAO activity is significantly reduced in inflamed or ulcerated mucosa in both Crohn’s disease and ulcerative colitis. The reduction correlates with histological injury, especially loss of mature enterocytes—the main source of DAO [8,13]. DAO is therefore understood not as a primary cause of inflammation but as a marker of enterocyte loss and mucosal damage [8,10,13].

Simultaneously in IBD, increased histamine release from mast cells and basophils leads to local accumulation of histamine within the intestinal mucosa and gut lumen [88,89,90]. Excess histamine activates H1 and H2 receptors, contributing to clinical manifestations typical of active IBD, including diarrhea, motility disturbance, abdominal pain and increased intestinal permeability [62]. Studies have demonstrated that histamine metabolism is markedly upregulated in active IBD and that mast cell activation is enhanced during active inflammation, promoting increased histamine turnover and increased formation of its major metabolite, N-methylhistamine (NMH) [91]. Accordingly, urinary NMH excretion was markedly higher in active CD and UC than in remission or in healthy controls. Moreover, urinary NMH concentrations correlated with the Crohn’s Disease Activity Index (CDAI), the Colitis Activity Index (CAI), C-reactive protein, and the severity of gastrointestinal symptoms [91]. Histamine may exert pro-inflammatory effects in IBD through direct modulation of immune response [71,92]. Several studies have demonstrated that histamine can stimulate the production of pro-inflammatory cytokines, including interleukin-6 (IL-6) and tumour necrosis factor (TNF), by intestinal epithelial cells and immune cells [45,92,93]. Consequently, an excess of histamine in the intestinal mucosa or lumen may not simply be a bystander effect of inflammation, but an active contributor sustaining chronic inflammatory processes. Nevertheless, the reduction in intestinal DAO activity due to enterocyte damage may amplify the local effects of histamine, exacerbating symptoms and barrier dysfunction in active IBD [11,12].

Although mucosal DAO is consistently reduced in IBD due to enterocyte loss, serum DAO activity may paradoxically increase in a subset of patients with active Crohn’s disease. This apparent discrepancy can be explained by the fact that severe mucosal injury leads to increased permeability, allowing intracellular enzymes, including DAO, to leak from damaged enterocytes into the bloodstream. Such leakage is often accompanied by other elevated circulating markers of barrier dysfunction, indicating that higher serum DAO does not reflect enhanced enzymatic synthesis but rather the extent of epithelial disruption [94]. Importantly, elevated serum DAO and increased urinary N-methylhistamine (NMH) reflect different aspects of the pathophysiology of active IBD [91]. While increased serum DAO is considered a marker of epithelial injury resulting from passive leakage from damaged enterocytes, elevated NMH primarily reflects increased histamine turnover driven by enhanced histamine release during intestinal inflammation [91,94]. Therefore, these observations are complementary rather than contradictory. Studies have reported that serum DAO is significantly elevated in patients with IBD and shows a positive correlation with disease activity. They also demonstrated that DAO outperformed traditional inflammatory markers such as C-reactive protein (CRP) in identifying depressive symptoms among individuals with IBD and highlighted a potential role of DAO as a biomarker reflecting disturbances within the gut–brain axis [14]. Importantly, serum DAO appears to capture different physiological processes and may not parallel mucosal levels. This divergence underscores the need to interpret serum and tissue DAO as distinct biomarkers, each capturing a separate facet of intestinal pathophysiology. It is conceivable that circulating DAO reflects the extent of epithelial barrier disruption and could theoretically be modulated by hepatic clearance, intestinal perfusion or the timing and severity of tissue injury.

### 6.2. Intestinal Dysbiosis

The intestinal concentration of histamine is tightly regulated by the composition and metabolic capacity of the gut microbiota, which collectively determine the balance between its synthesis and degradation. Microbiota-driven histamine formation depends largely on bacterial histidine decarboxylase activity, which converts luminal L-histidine into histamine and thereby increases local histamine burden. Reported histamine-producing taxa include *Enterobacteriaceae*, selected *Lactobacillus* strains (e.g., *L. reuteri*, *L. vaginalis*), as well as representatives of *Enterococcus*, *Staphylococcus*, *Proteus*, and *Clostridium* (including *C. perfringens*) [30]. Among these, *Lactobacillus reuteri* has been particularly well characterized for its probiotic and histaminogenic properties. This species expresses the eriC proton-chloride antiporter, which facilitates histidine decarboxylation and histamine release. The resulting bacterial histamine exhibits immunoregulatory effects by downregulating tumour necrosis factor (TNF) synthesis in myeloid cells [95]. Moreover, *Lactobacillus reuteri*-derived histamine has been shown to attenuate other pro-inflammatory cytokine pathways (IL-6, IL-22, TNF, IL-β) through activation of the H_2_ receptor, and protects against inflammation-driven colorectal tumorigenesis in vivo [96,97,98,99,100]. In contrast, intestinal overgrowth of histamine-producing species such as *Staphylococcus* spp., *Proteus* spp. or *Clostridium perfringens* can lead to excessive luminal histamine accumulation, particularly in individuals with reduced diamine oxidase activity, predisposing to histamine intolerance (HIT) and mucosal inflammation [30]. Studies have demonstrated that patients with histamine intolerance (HIT) exhibit a marked increase in bacteria belonging to the phylum Proteobacteria, a microbial signature widely recognized as indicative of dysbiosis and heightened inflammatory stress within the gut. Moreover, these patients showed significantly elevated concentrations of zonulin, a biomarker of impaired intestinal barrier integrity, which facilitates the translocation of histamine into the bloodstream and contributes to the characteristic symptomatology of the disorder [101]. Histamine-degrading capacity in the gut depends largely on DAO expression by intestinal epithelial cells. DAO levels are sensitive to microbial composition. Increased DAO activity has been associated with the presence of beneficial taxa such as *Faecalibacterium prausnitzii*, which maintain mucosal integrity [102]. In their study, Shi et al. demonstrated that individuals with elevated serum DAO concentrations exhibit distinct microbial alterations: a depletion of *Bacteroides* and *Lachnospira* and an enrichment of *Blautia*, *Faecalibacterium* and *Pasteurella*, suggesting that DAO dysregulation reflects underlying intestinal barrier dysfunction and microbial imbalance, even in the absence of systemic inflammatory cytokine activation. Furthermore, probiotic strains such as *Brevibacterium sediminis* have demonstrated the ability to degrade histamine in vitro and in vivo, thereby reducing luminal histamine concentrations and promoting mucosal homeostasis [103]. More recently, selected probiotic strains have also been shown to modulate host DAO secretion. In an in vitro study, *Lactiplantibacillus plantarum* LP115 increased DAO release from human intestinal epithelial HT-29 cells while reducing extracellular histamine concentrations, suggesting an additional mechanism by which probiotics may contribute to histamine homeostasis. However, these findings require confirmation in clinical studies [104]. Notably, microbiota-derived histamine engages epithelial H_2_ and H_4_ receptors, modulating local immune responses. Collectively, these findings underscore that the histamine-DAO-microbiota axis plays a pivotal role in maintaining intestinal immune equilibrium. The dynamic interplay between histamine-producing and histamine-degrading bacteria, in concert with epithelial DAO activity, regulates mucosal permeability and cytokine tone.

### 6.3. Intestinal Ischemia

Evidence for the role of DAO in intestinal ischemia is derived from both human and experimental studies. In human small intestinal tissue, ischemic injury has been shown to cause a marked decrease in mucosal DAO activity [105]. Most subsequent evidence comes from animal models. In rat models, plasma DAO increases rapidly after the onset of ischemia, reflecting leakage of the enzyme from injured enterocytes into the circulation [106]. Another rat study shows that serum DAO rises within minutes of superior mesenteric artery occlusion and correlates strongly with histological injury severity (r = 0.909, *p* < 0.01), with values exceeding the diagnostic threshold of 29.8 U/L providing a sensitivity of over 94% and a specificity of 100% for early ischemia detection [107]. In rat plasma samples, the combined assessment of DAO and citrulline (reflecting functional enterocyte mass), characterized by elevated DAO and reduced citrulline levels, enhances the diagnostic accuracy for acute mesenteric ischemia while reflecting the severity of mucosal necrosis and reperfusion injury [108]. DAO kinetics differentiate simple mechanical bowel obstruction, characterized by increased serum DAO, from strangulated obstruction with ischemia, in which both tissue and plasma DAO decrease sharply and are accompanied by elevated IL-6 and TNF-α levels, in a rat small intestine model [109]. Rabbit studies likewise demonstrate that circulating DAO declines within one hour of experimental ischemia, reflecting severe enterocyte loss [110]. In rabbit hemorrhagic shock models, DAO elevation correlates with both intestinal permeability and pro-inflammatory cytokine surges, further confirming its association with mucosal injury and systemic inflammation [111]. Finally, a recent meta-analysis of preclinical animal studies confirms that therapeutic interventions, including mesenchymal stem cell therapy, attenuate DAO alterations following intestinal ischemia–reperfusion injury, indicating preservation of mucosal integrity [112]. In summary, DAO is a rapid, quantitative biomarker that reflects the degree of intestinal mucosal injury, useful for early diagnosis, differentiation of ischemic lesions, and monitoring therapeutic efficacy in acute mesenteric ischemia.

### 6.4. DAO and Histamine in Functional Gastrointestinal Disorders

Increasing evidence suggests that altered histamine handling, together with reduced diamine oxidase (DAO) capacity, may represent one of the mechanistic contributors to functional gastrointestinal disorders (FGIDs). In this context, histamine signalling intersects with key mechanistic domains implicated in FGIDs, including visceral hypersensitivity, epithelial barrier dysfunction, and alterations in gastrointestinal motility [4]. In irritable bowel syndrome (IBS), histamine may serve as an effector mediator linking mucosal immune activation with symptom generation, particularly abdominal pain. Within IBS, histamine-related mechanisms have been linked to abdominal pain via neuro-immune interactions. In particular, symptom intensity has been associated with mucosal mast cell activation occurring in the vicinity of enteric nerve fibres [113]. In line with increased mucosal mediator release, biopsy-derived measurements indicate higher histamine levels in IBS patients than in healthy controls [114]. Receptor-level modulation may further contribute to this phenotype. Increased expression of histamine receptors, particularly H_1_R and H_2_R, has been reported in IBS and may facilitate enhanced sensory signalling in response to histamine [114]. Together, these data support a model in which excess histamine acts as a sensitizing mediator, promoting visceral hypersensitivity and symptom fluctuations. Importantly, clinical pharmacology provides indirect support for the role of histamine-driven pathways in FGIDs. Mast cell stabilizers have demonstrated symptomatic benefit in subsets of patients. Disodium cromoglycate has been associated with reduced abdominal pain and diarrhea in a subset of patients with functional dyspepsia and IBS, and experimental data further suggest that cromones may attenuate stress-associated increases in intestinal permeability [115,116]. Similarly, ketotifen has been shown to reduce visceral hypersensitivity, reinforcing a contribution of mast cell-derived mediators, including histamine, to symptom generation [117,118]. While mast cell-targeted therapies have shown benefits in some FGID phenotypes, histamine receptor signalling itself may represent an independent therapeutic target. In IBS, H1 receptor antagonists have been associated with symptom improvement in selected cohorts, supporting the relevance of histamine-driven sensitization mechanisms [37,118,119]. In parallel, microbiome-related mechanisms have been increasingly implicated in the regulation of histamine availability in IBS. Metagenomic data suggest enrichment of histidine decarboxylase (HDC)-positive bacteria in selected IBS phenotypes, which may increase microbial histamine production [120]. Among the taxa reported to be more common in IBS are organisms such as Enterobacteriaceae and *Clostridium perfringens*, which are capable of decarboxylating histidine, potentially increasing luminal histamine levels [120,121,122,123]. Clinically, dietary triggers are frequently reported in IBS, and symptom exacerbation after ingestion of histamine-rich foods appears common, suggesting that luminal histamine exposure may interact with mucosal susceptibility mechanisms [124]. In this context, low serum DAO activity has been described in patients with chronic abdominal symptoms and food intolerance, often co-occurring with lactose or fructose malabsorption, raising the hypothesis that impaired histamine degradation may constitute a shared mechanistic component in selected subgroups [125]. Experimental work further suggests that enzymatic approaches may modify histamine-related motor responses. Preclinical data suggest that vegetal diamine oxidase (vDAO) attenuates histamine-induced contractions of murine distal colon smooth muscle more effectively than conventional antihistamines. Pyridoxal 5′-phosphate has been identified as a cofactor that enhances the antispasmodic effect of vDAO [11]. Collectively, these findings support the concept that impaired DAO activity, increased histamine signalling, and dysbiosis-related histamine production may act synergistically to drive hypersensitivity, dysmotility, and barrier disturbances in FGIDs. Therapeutic strategies aimed at restoring histamine balance, through DAO supplementation, dietary histamine restriction or microbiota modulation, may therefore represent promising approaches to alleviate symptoms and restore mucosal homeostasis [48,126].

### 6.5. DAO Alterations in Other Gastrointestinal Disorders

Beyond intestinal ischemia, diamine oxidase activity fluctuates across various gastrointestinal conditions. In celiac disease, both mucosal and serum DAO activity is markedly decreased due to villous atrophy and enterocyte loss, with partial recovery following adherence to a gluten-free diet [127]. Profound DAO suppression is also observed in chemotherapy-induced mucositis, where cytotoxic agents such as cytarabine or 5-fluorouracil induce mucosal apoptosis and crypt loss, leading to DAO reductions to <10% of baseline levels in both plasma and intestinal tissue [9,16,128]. Moreover, Miyoshi et al. demonstrated that a decline in serum DAO activity preceded the onset of diarrhea in all patients, and the extent of this decrease correlated significantly with both diarrhea severity and the reduction in duodenal villus height and surface area from baseline [16]. Similarly, in radiation-induced enteropathy, experimental studies demonstrate a significant decline in ileal tissue and circulating DAO after abdominal irradiation [129]. DAO insufficiency has also been associated with various forms of carbohydrate malabsorption [78]. In fact, individuals with lactose intolerance who simultaneously exhibit reduced plasma DAO activity tend to show markedly elevated end-expiratory hydrogen values and report a greater number of symptoms during the hydrogen breath test compared with lactose-intolerant patients whose DAO levels remain within the normal range [130]. DAO levels are also significantly reduced in anorexia nervosa and malnutrition, reflecting mucosal atrophy and reduced enterocyte regeneration [131], as well as in gastrointestinal malignancies where decreased DAO activity in tumour-adjacent mucosa signifies dedifferentiation and impaired mucosal turnover [132]. DAO alterations also occur in food allergies and histamine-related food hypersensitivity, where low mucosal DAO leads to impaired histamine catabolism and increased intestinal inflammation [133]. Recent investigations have highlighted a possible association between diminished DAO activity and non-celiac gluten sensitivity (NCGS) [129,134]. Notably, the study by Griauzdaitė et al. reported that the vast majority, approximately 90% of individuals diagnosed with NCGS, exhibited reduced serum DAO activity [79].

### 6.6. DAO in Liver Disorders

Circulating DAO levels are markedly elevated in individuals with liver cirrhosis compared with healthy controls, a finding that mirrors enhanced intestinal permeability and the presence of systemic endotoxemia. Pioneering investigations by Ruan and colleagues revealed concomitant elevations of plasma DAO, D-lactate, and endotoxin in cirrhotic patients, with strong inter-parameter correlations, providing evidence of simultaneous intestinal barrier impairment and augmented bacterial translocation. Notably, serum DAO activity tends to be higher in patients with Child-Pugh class A and B disease, whereas a decline is observed in Child-Pugh class C, most plausibly reflecting advanced mucosal villous atrophy, enterocyte loss, and diminished enzymatic production [135]. Zhang et al. reported that metabolic dysfunction-associated fatty liver disease (MAFLD) is characterized by compromised intestinal barrier integrity, with circulating diamine oxidase and D-lactate proposed as potential indicators of the disease, whose serum concentrations are associated with the degree of hepatic steatosis and metabolic disturbances observed in affected patients [136]. In HBV-related decompensated cirrhosis, higher serum DAO activity independently predicts six-month readmission and recurrence of hepatic encephalopathy, performing better than conventional prognostic indices such as the Child-Pugh score [137]. Beyond cirrhosis, DAO alterations have been observed during orthotopic liver transplantation, where baseline DAO levels are markedly higher in end-stage liver disease patients than in healthy subjects, and rise sharply during the anhepatic and reperfusion phases. These fluctuations inversely correlate with histamine concentration and positively with norepinephrine requirements, suggesting that intraoperative DAO dynamics reflect systemic vasoregulatory and intestinal responses rather than hepatic metabolism [138]. Similarly, plasma DAO serves as an independent prognostic indicator in acute-on-chronic hepatitis B liver failure, where elevated DAO concentrations predict one-month mortality more accurately than the MELD score, underscoring DAO’s prognostic value in hepatic decompensation [139]. Recent studies exploring cirrhosis-associated gut dysbiosis have further implicated DAO in host-microbiota interactions, demonstrating that lower circulating DAO levels are associated with more profound microbial imbalance and more advanced Child-Pugh stage [140].

Figure 1 summarizes the major determinants of DAO activity. Alterations in any of these domains may impair histamine degradation, contribute to intestinal barrier dysfunction, and promote histamine-related symptoms and systemic effects. Examples of drugs reported to inhibit DAO activity include verapamil, clavulanic acid, isoniazid, and diclofenac [71,72,73,74].

## 7. Conclusions

Diamine oxidase (DAO) plays a pivotal role in maintaining extracellular histamine homeostasis within the gastrointestinal tract, and its altered activity is increasingly implicated in a broad range of gastrointestinal disorders. Reduced mucosal DAO reflects epithelial injury, impaired barrier integrity, and increased histamine signalling, while transient elevations in serum DAO often indicate acute mucosal leakage. Although these features highlight DAO as a sensitive indicator of intestinal health, its substantial physiological and pathophysiological variability, shaped by genetics, inflammation, dysbiosis, nutrition, and medication exposure, limits its reliability as a standalone diagnostic marker. Nonetheless, emerging data support the potential of DAO as an adjunctive biomarker and therapeutic target in conditions such as histamine intolerance, IBD, functional GI disorders, liver disease, and intestinal ischemia. Future progress needs standardized measurement methods, validated reference ranges, and integrative approaches that combine DAO metrics with clinical, genetic, and microbiome-based assessments.

## Data Availability

No new data were created or analyzed in this study. Data sharing is not applicable to this article.
